# Supplementary material for: Characterizing the Core-Shell Architecture of Block Copolymer Nanoparticles with Electron Microscopy: A Multi-Technique Approach
Source: Polymers (Basel). 2020 Jul 25;12(8):1656. doi: 10.3390/polym12081656 (PMC7464915; doi:10.3390/polym12081656)
Supplement: Supplementary file 1 [file polymers-12-01656-s001.pdf]

polymers

Supplementary Information

Characterizing the Core-shell Architecture of Amphiphilic Block Copolymer Spherical Nanoparticles: A Multi-Technique Approach

Vitalii Tkachenko[1,2], Loïc Vidal[1,2], Ludovic Josien[1,2], Marc Schmutz[3], Julien Poly[1,2] and Abraham Chemtob[1,2*]

[1] Université de Haute-Alsace, CNRS, IS2M UMR7361, F-68100 Mulhouse, France
[2] Université de Strasbourg, France
[3] Université de Strasbourg, CNRS, ICS UPR22, F-67034 Strasbourg, France
* Correspondence: +33 3 8960 8834, abraham.chemtob@uha.fr

Received: 26 June 2020; Accepted: 17 July 2020; Published: 25 July 202

Synthesis Protocol 1
Additional Figures 2
Additional Table 7
References 8

Synthesis Protocol

Synthesis protocol of diblock copolymer nanoparticles *via* photomediated dispersion PISA of St (ex. poly(hydroxyethyl acrylate)-*b*-polystyrene – $PHEA_{85}$-*b*-PS_{130}). In a typical synthesis, $PHEA_{85}$-TTC (0.838 g, 0.083 mmol) (TTC – S-cyanomethyl-S-dodecyltrithiocarbonate), Styrene (1.721 g, 16.528 mmol) and a methanol/water mixture (8.177 g/0.430 g, 95/5 w/w %) were charged into a Schlenk tube equipped with a stirring bar. The mixture was degassed by three freeze–pump–thaw cycles before being placed under nitrogen. The tube was then placed in a pre-heated oil bath at 35 °C and irradiated during 70 h in an immersion type photoreactor (λ = 472 nm, 547 mW cm^{-2}).

PS latex was prepared using semi-continuous emulsion polymerization. In this method, surfactant (SDS, 0.2024 g, 0.701 mmol) and initiator (ammonium persulfate, 0.0264 g, 0.116 mmol) were dissolved in water (40 g) and added to a glass reactor (Schlenk tube) equipped with a stainless steel stirrer, a reflux condenser, a sampling device, a nitrogen gas inlet tube, and a temperature probe. When the reaction temperature was reached (80 °C), the monomer (8.8 g, 84.5 mmol) was continuously fed (49 mg min^{-1}) over a period of 3 h. At the end of monomer feeding, the system was maintained at the reaction temperature for 60 min to minimize the amount of residual monomer [1].

Additional Figures

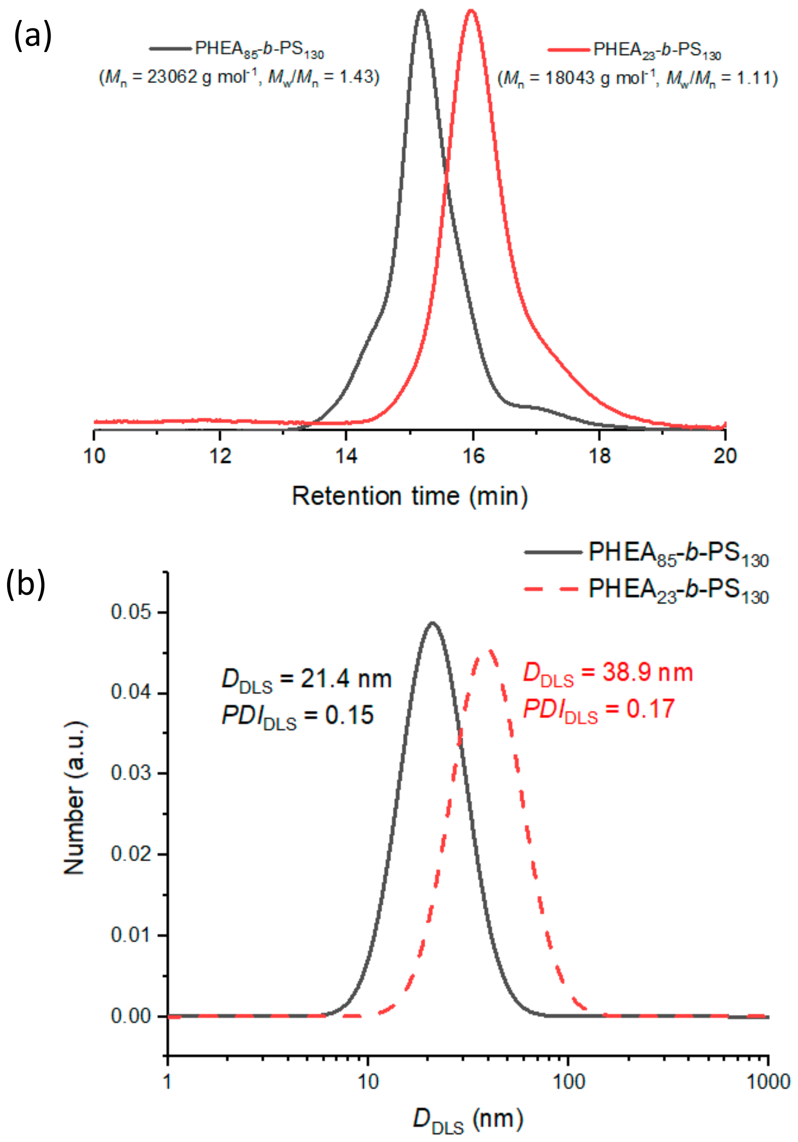

Figure S1. (a) SEC traces in DMF at 50 °C (refractive index detector) of PHEA$_{85}$-*b*-PS$_{130}$ and PHEA$_{23}$-*b*-PS$_{130}$ diblock copolymers. (b) Number-weighted size distributions (DLS data, cumulants method) of copolymer nanoparticles in MeOH/water (95/5 %vol.).

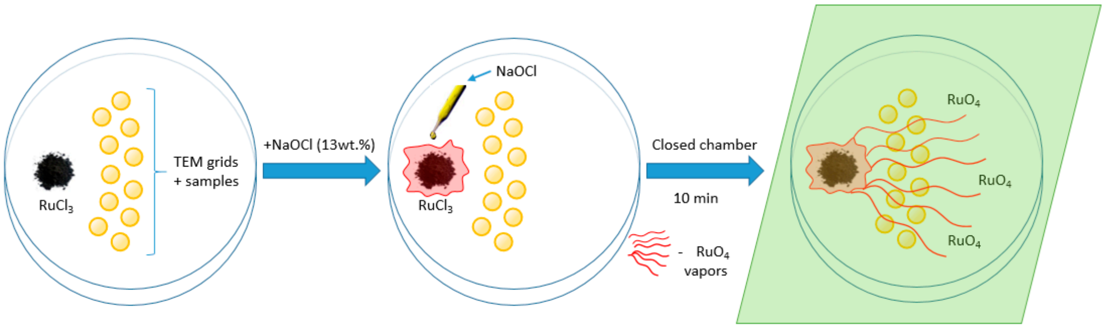

Figure S2. Schematic representation of positive staining technique and set-up.

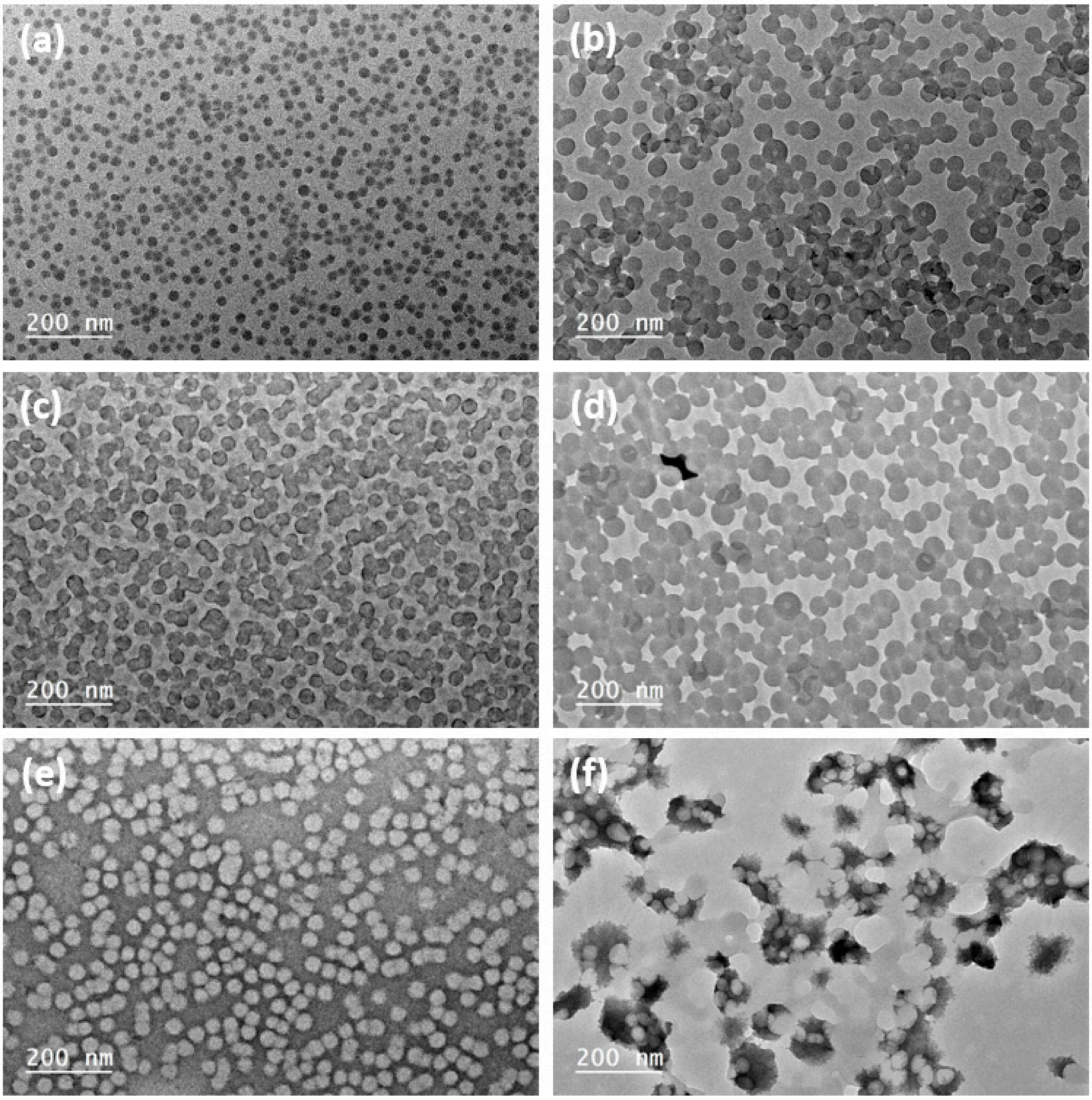

Figure S3. Low magnification TEM images of PHEA$_{85}$-*b*-PS$_{130}$ (left column: a, c, e) and PHEA$_{23}$-*b*-PS$_{130}$ (right column: b, d, f) nanoparticles. The first line is without staining, the second after RuO_4 positive staining, the third after negative staining.

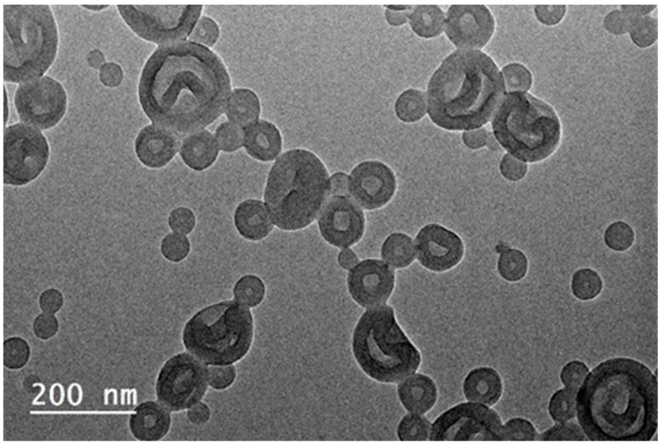

Figure S4. TEM image of vesicular nano-objects derived from air-dried unstained PHEA$_{17}$-*b*-PS$_{136}$ copolymer.

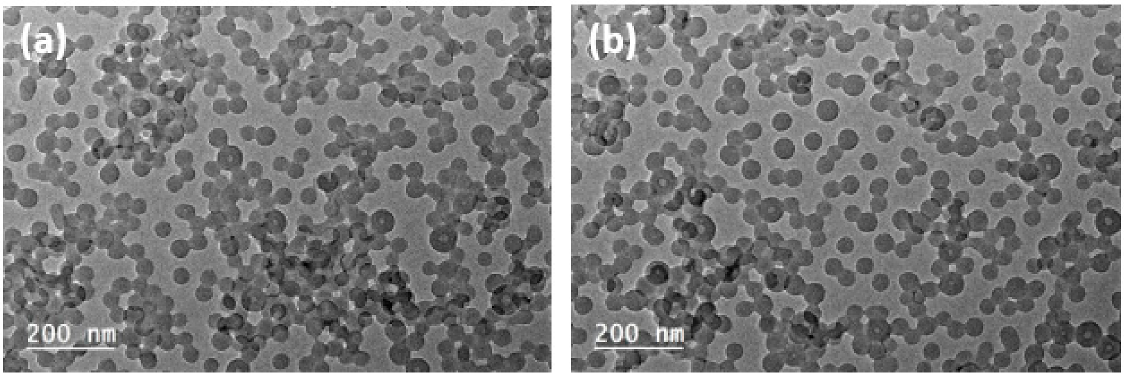

Figure S5. TEM images of air-dried unstained PHEA$_{23}$-*b*-PS$_{130}$ block copolymer nanoparticles prepared at different concentrations: (**a**) 1 wt% and (**b**) 0.2 wt%.

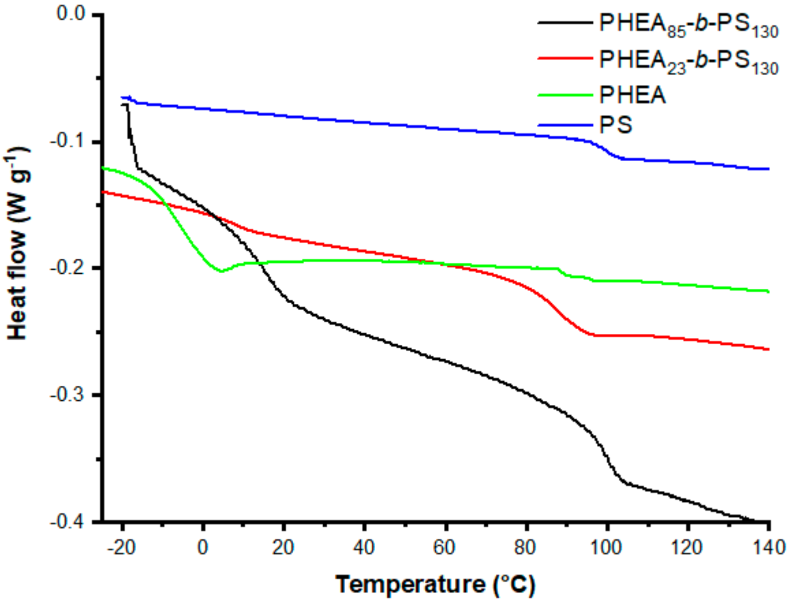

Figure S6. DSC traces of PHEA$_x$-*b*-PS$_y$ diblock copolymer and individual blocks. Experimental conditions are detailed in **Table S1**.

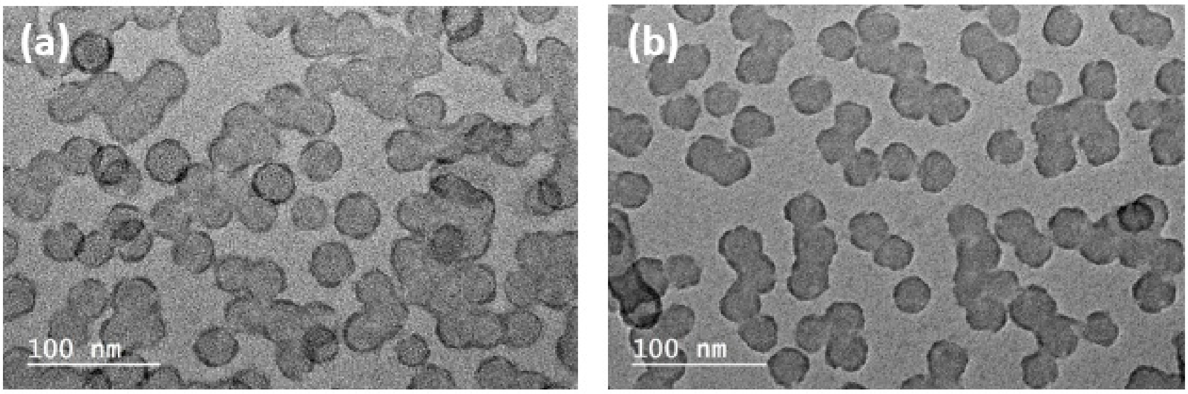

Figure S7. Effect of dialysis on TEM images: (a) without and (b) dialyzed PHEA$_{85}$-*b*-PS$_{130}$ reaction mixture with subsequent RuO_4 staining.

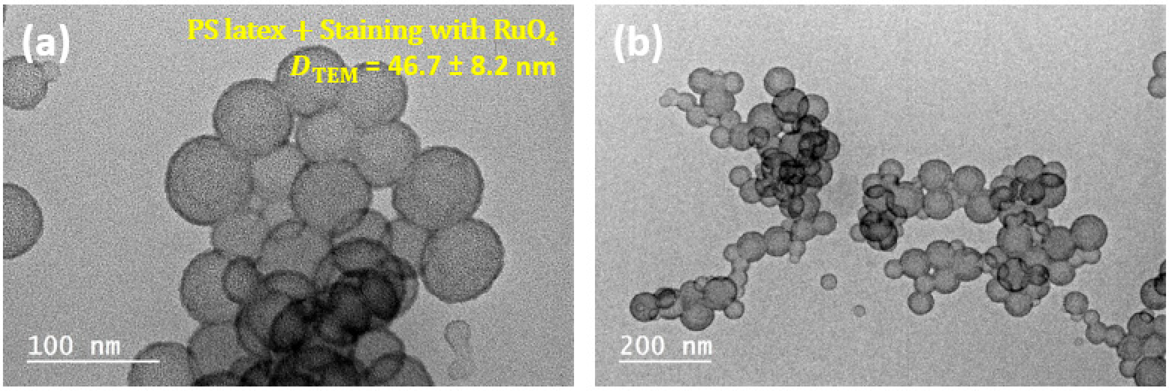

Figure S8. (a) TEM image of air-dried PS latex synthesized *via* feeding mode [1] stained with RuO_4. (b) Corresponding low-magnification image.

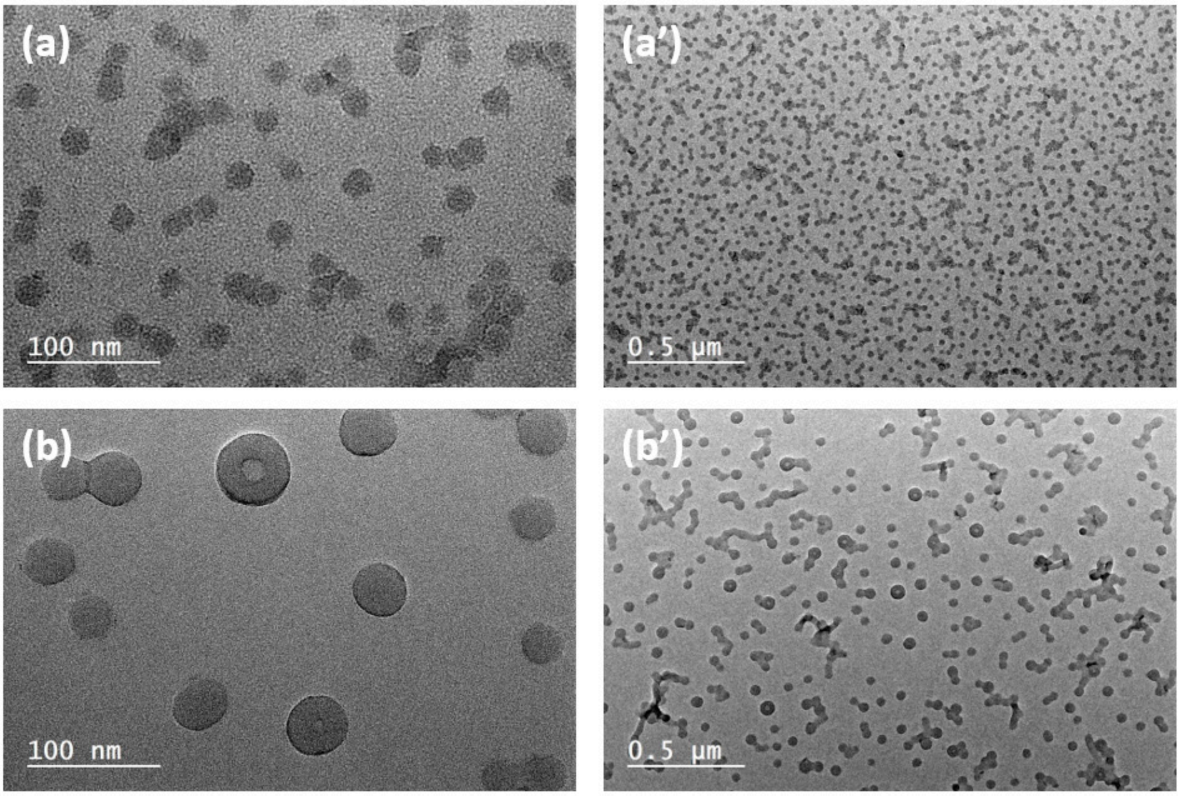

Figure S9. Positive stained TEM image of (**a**) $PHEA_{85}$-*b*-PS_{130} and (**b**) $PHEA_{23}$-*b*-PS_{130} with OsO_4. (a', b'). Corresponding low magnification images.

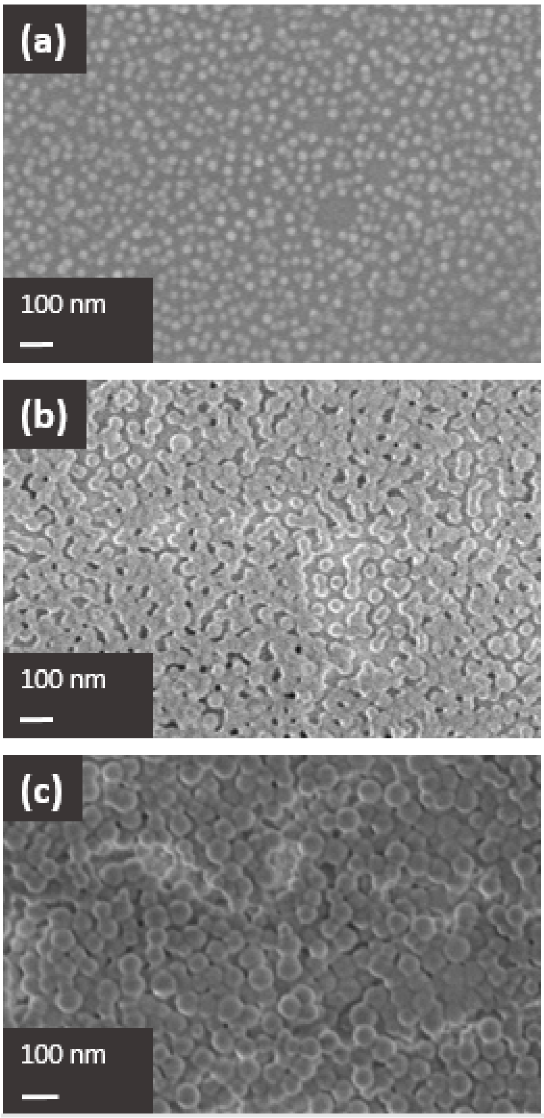

Figure S10. SEM images of (a) $PHEA_{85}$-*b*-PS_{114}, (b) $PHEA_{25}$-*b*-PS_{118} diblock copolymer based nanoparticles and (c) PS latex.

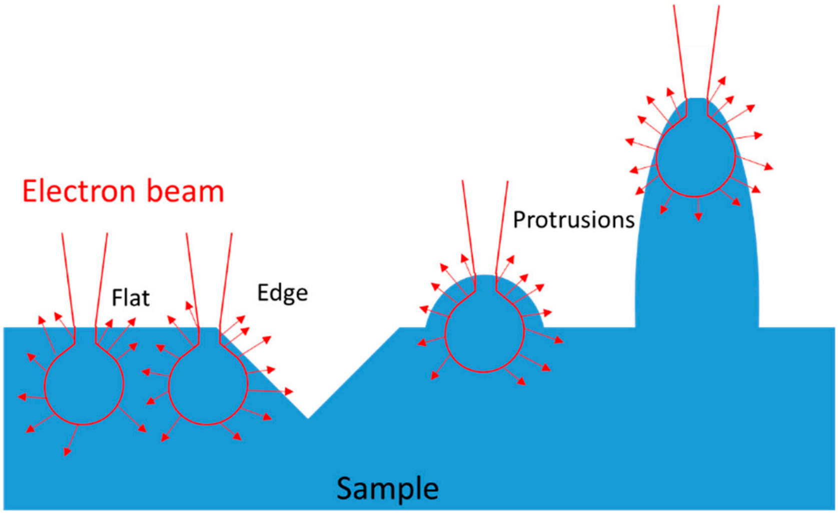

Figure S11. Schematic representation of the edge effect inspired from ref [2]. Particle edge or protrusions at particle surface appear brighter than plain surface through the escape of a larger number of secondary electrons.

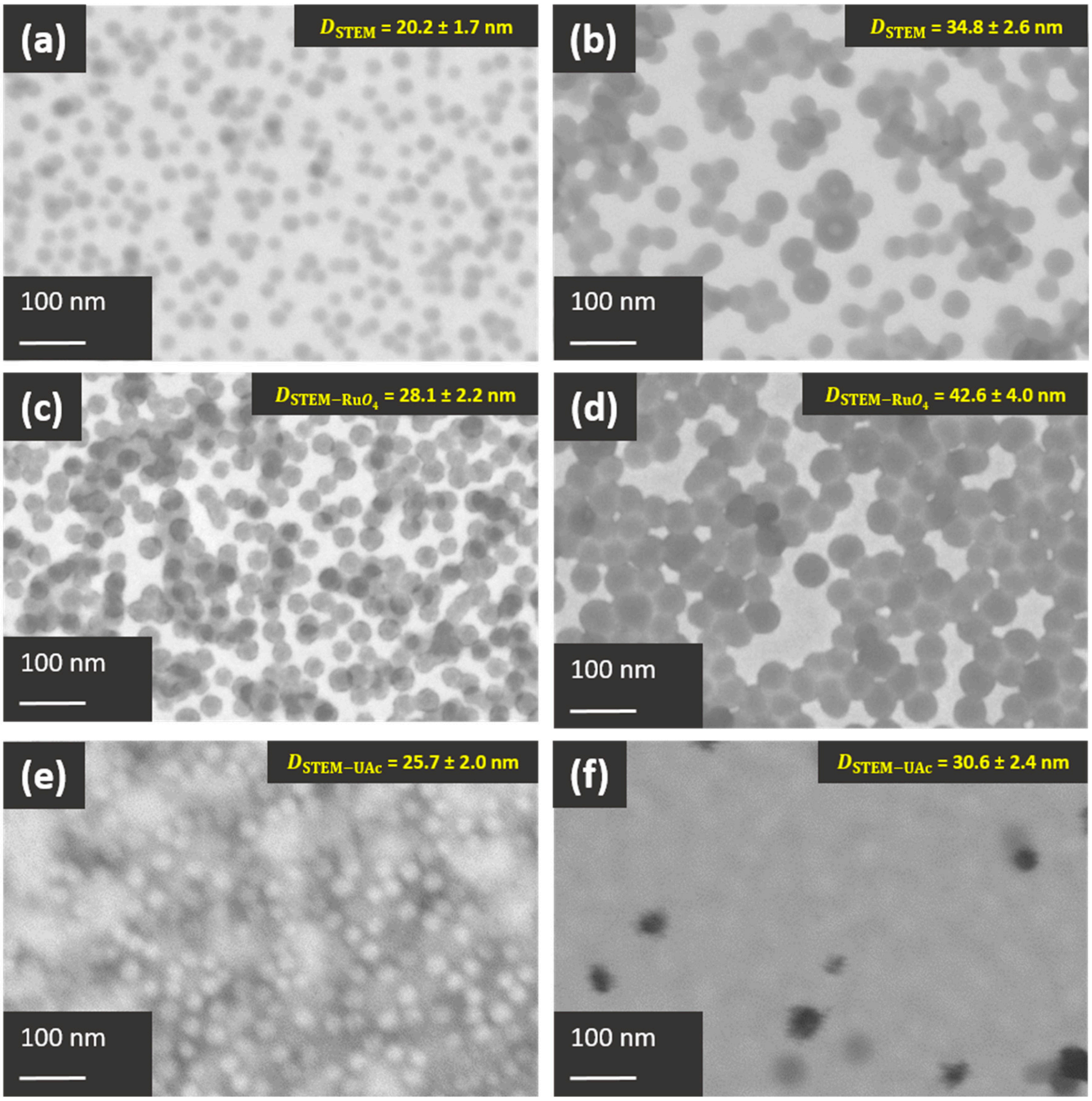

Figure. S12. STEM images of (a) $PHEA_{85}$-*b*-PS_{114} and (b) $PHEA_{25}$-*b*-PS_{118} diblock copolymer based nanoparticles. STEM images with RuO_4 positive staining of (c) $PHEA_{85}$-*b*-PS_{114} and (d) $PHEA_{25}$-*b*-PS_{118} diblock copolymer based nanoparticles. STEM images with UAc negative staining of (e) $PHEA_{85}$-*b*-PS_{114} and (f) $PHEA_{25}$-*b*-PS_{118} diblock copolymer based nanoparticles.

Additional Table

Table S1. Glass transition temperatures determined by differential scanning calorimetry. The copolymer and pure phase samples were heated from −50 to 150 °C, cooled to −50 °C, and reheated to 150 °C. The heating and cooling rates were ±10 °C/min. Glass transition temperatures were determined during the third heating cycle.

System	T_g^{PHEA}, °C	T_g^{PS}, °C
$PHEA_{23}$-*b*-PS_{130}	10.7	98.7
$PHEA_{85}$-*b*-PS_{130}	7.0	87.7
PHEA	-7.3	-
PS	-	99

T_g^{PHEA} – Glass transition temperature of PHEA phase, T_g^{PS} – Glass transition temperature of PS phase.

References

1. Nunes, J. de S.; Asua, J.M. Theory-Guided Strategy for Nanolatex Synthesis. *Langmuir* **2012**, *28*, 7333–7342, doi:10.1021/la3006647.
2. Ul-Hamid, A. *A Beginners' Guide to Scanning Electron Microscopy*; Springer International Publishing: Cham, 2018; ISBN 978-3-319-98481-0.
